# Feeding Byproduct-Based Concentrates Instead of Human-Edible Feed Ingredients Increases Net Food Production and Improves Performance of High-Producing Holstein Cows

**DOI:** 10.3390/ani12212977

**Published:** 2022-10-29

**Authors:** Nima Naderi, Gholam Reza Ghorbani, Hamid Erfani, Luiz Felipe Ferraretto

**Affiliations:** 1 Department of Animal Sciences, College of Agriculture, Isfahan University of Technology, Isfahan 8415683111, Iran; 2 Department of Animal and Dairy Science, University of Wisconsin-Madison, Madison, WI 53706, USA

**Keywords:** by-product, human-edible feed ingredients, net food production, dairy cow

## Abstract

**Simple Summary:**

The effect of replacing human-edible feed ingredients with byproducts on the performance and net food production of high-producing Holstein dairy cows was investigated. Feeding byproduct-based concentrate instead of human-edible feed ingredients increased net food production and improved the performance of high-producing Holstein cows.

**Abstract:**

The effect of feeding greater amounts of byproducts (BP) as a replacement for human-edible (HE) feed ingredients on nutrient intake, chewing activity, rumen fermentation, production performance, human-edible feed conversion efficiency (HeFCE) and net food production (NFP) of high-producing Holstein cows was evaluated. Twelve multiparous Holstein cows (BW = 673 ± 44, DIM = 112 ± 8 d; 48 ± 2.25 kg/d of milk; mean ± SE) were used in a replicated 3 × 3 Latin square design with 28-d periods. Each period consisted of 21 d of adaptation followed by 7 d of data collection. Treatments diets were (DM basis): (1) concentrate containing 26% byproducts (BP26; control); (2) concentrate containing 60% byproducts (BP60); and (3) concentrate containing 95% byproducts (BP95). Alfalfa hay (20% dietary DM) and corn silage (20% dietary DM) were included in all diets. Dietary concentrations of neutral detergent fiber (NDF), non-fiber carbohydrates (NFC), starch and ether extract (EE) were 32.1, 41.0, 26.14 and 3.4% (BP 26); 35.3, 36.0, 22.05 and 4.7% (BP60); and 38.2, 32.0, 17.96 and 6.1% (BP95), respectively (DM basis). Dry matter (22.07 kg/d) and NEL (35.16 Mcal/d) intakes did not differ among treatments. However, ether extract and NDF intakes increased, whereas starch intake decreased linearly as BP ingredients increasingly replaced HE feed ingredients. Eating time was not affected by dietary treatment, but ruminating and total chewing time tended to increase with increasing amounts of BP. Replacing HE with BP ingredients did not affect rumen pH. An increased proportion of BP ingredients in the diet linearly decreased propionate, isobutyrate, isovalerate and valerate concentrations in the rumen and increased acetate concentration and the acetate to propionate ratio. Replacing HE with BP ingredients did not affect milk yield. The yield of 3.5% FCM (39.12, 40.14 and 41.33 kg/d for BP26, BP60 and BP95, respectively) and fat content (2.95, 2.99 and 3.13 % for BP26, BP60 and BP95, respectively) linearly increased. Substituting BP ingredients for HE feed ingredients increased unsaturated fatty acids, monounsaturated fatty acids, polyunsaturated fatty acids, stearic acid, oleic acid and preformed fatty acids but decreased saturated fatty acids, palmitic acid, de novo and mixed fatty acids. Replacing HE with BP feed ingredients increased human-edible efficiency (HeFCE) for crude protein (1.06, 1.66 and 4.14 kg/kg edible for BP26, BP60 and BP95, respectively) and for energy (2.27, 3.62 and 9.22 MJ/MJ edible for BP26, BP60 and BP95, respectively) and also net food production (NFP) for crude protein (0.064, 0.52, and 1.00 kg/d for BP26, BP60, and BP95, respectively) and energy (62.8, 83.0 and 104.7 MJ/d for BP26, BP60 and BP95, respectively). Feeding byproduct-based concentrates instead of human-edible feed ingredients increase human-edible feed conversion efficiency (HeFCE), net food production (NFP) and improved the performance of high-producing Holstein cows.

## 1. Introduction

Request for livestock products will increase in the future due to population growth and a greater per capita income [1]. Because of the limited probabilities of increasing the area of arable land, the increased requests for livestock feeds depends on an increase in crop yield per hectare. There is competition for existing arable land among food, feed and fuel. Considering that more than 70% of global agricultural land is already being used for livestock feed production [2], feeding less human-edible (HE) ingredients to animals is essential. Lindberg et al. (2021) reported that feeding a byproduct-based concentrate (BP) needed 35% less cropland, decreased carbon footprint by 20% and lowered eutrophication potential by 20% compared with feeding a cereal grain-based concentrate [3]. Therefore, feeding more BP from the human food, fiber and bio-fuel industries in the diet of dairy cows as a replacement of HE ingredients is a more sustainable approach.

Wilkinson [4] showed that dairy production is the most efficacious animal production system in the United Kingdom based on the ratio of human-edible input to output. The net food production (human-edible output minus human-edible input) was presented by Ertl et al. (2016) as a metric for human-edible feed conversion efficiency (HeFCE) [5]. Byproduct-based concentrates increase the net food production of dairy products compared with cereal grain and pulses in organic production [5,6,7,8,9].

Dairy farms feeding forages of moderate to low quality require diets formulated with high amounts of concentrate to meet the energy requirements of high-producing cows. Esmaeili et al. (2016) reported that the average usage of concentrate in the diet of Iranian dairy farms is 65% of DM [10]. This approach increases the risk of subacute ruminal acidosis through the presence of unbearable amounts of starch degradation in the rumen, potentially reducing animal production and risking animal health [11]. In addition, a high-concentrate diet with insufficient forage NDF may increase the passage of undigested starch into the small intestine, increasing the chances of remaining undigested or causing hindgut fermentation [12,13,14]. All these scenarios may decrease the efficiency of milk production and income over the feed cost of dairy farms. Besides, grains and soybeans are ingredients in human food and the consumption of grains and soybean by dairy cows may increase the competition of human and dairy cows for food and negatively affect the cost and availability of grains [15,16]. Therefore, strategies for decreasing human-edible feeds from dairy diets without compromising dry matter intake or milk production are warranted and have been the subject of recently published studies [6,9,17,18]. Despite the challenges of feeding byproducts (i.e., risk of mycotoxin contamination, variation in nutrient composition between batches, etc.), studies have demonstrated that diets with relatively high proportions of byproducts can maintain or even improve animal performance [19]. To our knowledge, however, most studies evaluating this diet manipulation strategy have been conducted with moderate-producing dairy cows (27–32 kg/d) [6,7,8,9].

The objective of this study was to evaluate if feeding byproduct-based concentrates instead of human-edible feed ingredients improved the net food production of high-producing Holstein cows and their production performance. Therefore, a byproduct-based concentrate replaced the commonly used concentrate based on human-edible feed ingredients. It was hypothesized that under the conditions of the Iranian dairy production system, byproducts as supplements can improve net food production (NFP) without negative effects on feed intake, production performance and efficiency measures. To ensure that the lower starch and higher fat and fiber contents of the byproduct-based concentrate had no negative effects on feeding behavior and rumen health, feeding behavior, rumen pH and rumen fermentation were also analyzed.

## 2. Materials and Methods

The experiment was conducted at the dairy facilities of the Lavark Research Station (Isfahan University of Technology, Isfahan, Iran). All animal procedures were approved by the Animal Care and Use Committee of Arak University (Protocol #IR2020-08) following the guidelines outlined by the Iranian Council of Animal Care (1995).

### 2.1. Animals, Experimental Design and Treatments

Twelve multiparous Holstein cows (BW = 673 ± 44, DIM = 112 ± 8 d; 48 ± 2.25 kg/d of milk; mean ± SE; at trial initiation) were used in a replicated 3 × 3 Latin square design with 28-d periods. Each period consisted of 21 d of adaptation followed by 7 d of data collection. Cows were housed in individual stalls (4 × 4 m) within a roofed facility with open sides and clean wood shavings and sand were used for bedding and refreshed daily. Three dietary treatments were formulated to replace human-edible feed ingredients with byproducts (DM basis): (1) concentrate containing 26% byproducts (BP26; control); (2) concentrate containing 60 % byproducts (BP60); and (3) concentrate containing 95% byproducts (BP95). Diets were formulated to meet or exceed the Cornell Net Carbohydrate and Protein System (version 5.0) nutrient allowance for a lactating dairy cow weighing 650 kg and producing 45 kg/d of milk with 3.0% milk true protein and 3.2% milk fat (Table 1). Feed was supplied twice daily at 1000 h and 1800 h in amounts that allowed for 10% refusals. Forty percent of the daily allocation was provided at the morning feeding and 60% in the afternoon.

### 2.2. Feed Intake and Chemical Analysis

The TMR amounts offered and refused were measured daily for each cow during day 21 to 28 of each period and daily DMI for each cow was calculated. Corn silage DM was determined weekly for adjustments of as-fed amounts in the TMR. Representative samples of forages (pooled within period), treatment TMR (pooled by diet within period) and individual refusals (pooled by cow within period) were taken immediately before the morning feeding during the 7-d collection period. All samples were immediately frozen at −20 °C until they were analyzed.

After thawing, the DM concentration of composited samples of forages, TMR and refusals was determined by drying at 60 °C in a forced-air oven for 48 h and DM results were adjusted to 100 °C according to AOAC ( [22]; method 925.40). All samples were ground using a Wiley mill through a 1-mm screen (Arthur H. Thomas, Philadelphia, PA, USA) and analyzed for CP using the Kjeldahl method (Kjeltec 1030 Auto Analyzer, Tecator, Höganäs, Sweden; [22], method 955.04), ether extract (EE; [22], method 920.39), ash ([22]; method 942.05) and NDF using heat stable α-amylase (100 μL/0.5 g of sample) and sodium sulfite [23]. The starch concentration in feed and fecal samples was determined using the modified glucoamylase procedure described by Zhu et al. (2016) [24]. Residual organic matter (ROM) was calculated as %OM-%CP-%NDF-%Starch-%fat according to NASEM (2021) [20].

### 2.3. Particle Size Measurement and Chewing Activity

During day 21 to 28 of the study, TMR and orts were collected daily for a determination of particle size. Particle size was measured in triplicate using the Penn State Particle Separator, equipped with 3 sieves (19 mm, 8 mm, and 1.18 mm) and a bottom pan [25]. After sieving, samples were placed in a forced-air oven at 60 °C to determine the DM of each sieved fraction. The physically effective factor (pef) values were determined as the total proportion of DM retained on 2 sieves (pef _> 8_; [26]) or 3 sieves of the Penn State Particle Separator (pef _> 1.18_; [25]; Table 2), respectively. The geometric mean particle size of TMR and orts was calculated according to ASAE (1995) procedures [27].

On day 27 of each period, chewing activity was monitored visually for each cow for 24 h. Three people participated in the observation, where a single observer monitored the cows constantly for 4 h, after which they were replaced by a new observer. During the 24-h period, eating and ruminating activities were recorded every 5 min, where the observer required approximately 1 min to make observations for all the cows and the activity of each cow was assumed to persist for the entire 5-min interval between observations [28]. The total chewing time was calculated as the sum of ruminating and eating times.

### 2.4. Ruminal pH and Fermentation

On day 28 of each period, approximately 4 h after the morning feeding, ruminal fluid (approximately 5 mL) was sampled from the ventral sac via rumenocentesis [28]. The pH of the ruminal fluid was immediately determined using a portable digital pH meter (HI 8318; Hanna Instruments, Cluj-Napoca, Romania), calibrated at pH 4 and 7 at the start of each measuring day. Then, 2 mL of the collected sample was acidified with 0.4 mL of a 25% meta-phosphoric acid solution and frozen immediately at −20 °C until subsequent analysis for VFA. Ruminal fluid for VFA determination was subsequently thawed and centrifuged at 10,000× *g* at 4 °C for 20 min and analyzed via gas chromatography (Chrompack, model CP-9002; Chrompack International BV, Middelburg, the Netherlands) using a 50-m (0.32 mm i.d.) fused-silica column (CP-Wax Chrompack Capillary Column; Varian Inc., Palo Alto, CA, USA) and crotonic acid as the internal standard.

### 2.5. Milk Yield and Components, Body Weight and Back Fat Thickness

The cows were milked thrice daily at 01:00, 09:00 and 17:00 in a herringbone milking parlor. Milk yields were recorded and milk samples were collected during the 5-d sampling period. Samples were preserved with potassium dichromate and stored at 4 °C pending analysis. Milk samples were sent to Ideh Sazan Rojan Alvand Co. (Alborz, Iran) for fat, true protein, lactose, SNF, total solids, MUN, nonesterified fatty acids, BHB and fatty acid analyses using Fourier transform mid-infrared spectroscopy of CombiScope FTIR 600 HP (Delta Instruments, Drachten, the Netherlands). The yield of 3.5% fat corrected milk (FCM) = 0.432 × milk yield + 16.23 × fat yield, energy corrected milk (ECM) = 12.82 × fat yield + 7.13 × protein yield + 0.323 × milk yield, and solid corrected milk (SCM) = milk yield × [(12.24 × fat% × 0.01) + (7.1 × protein% × 0.01) + (6.35 × lactose% × 0.01) − 0.0345] were calculated according to NRC (2001) equations [29].

Cows were weighed at the beginning (day 1) and the end (day 28) of each period. Back fat thickness (BFT) measured using an ultrasonographic method [30] were determined at the beginning (day 1) and the end (day 28) of each period.

### 2.6. Calculations and Statistical Analyses

The proportion of potential human-edible feeds was calculated according to the broad classification of Wilkinson (2011) [4]. Human-edible feed conversion efficiency for CP and GE was calculated as the human-edible content in the milk that the cows produced divided by the potential human-edible content of the feeds that the cows consumed. Net food production (as MJ of GE/d and kg of CP/d) was calculated as the human-edible content in the milk minus the potential human-edible amount in the feed consumed, according to [5]. Data on the GE content of the feedstuffs were retrieved from the Feedipedia database [31] and data on the GE content of milk from the nutritional database of USDA (2016) [32].

Data were analyzed using the mixed model procedure of SAS (Proc Mixed; SAS Institute, 2002) to account for the effects of square, cow within square and treatment. Treatment, square and period were considered as fixed effects; cow within square was considered a random effect. The mathematical expression of the model is:

Yijkl = μ + Pi + Sj + C(S)kj + Tl + eijkl,(1)

where Yijkl = the dependent variable, μ = the population mean, Pi = the fixed effect of period i, Sj = the fixed effect of square j, C(S)kj = the random effect of cow k within square j, Tl = the fixed effect of treatment l and eijkl = the random residual error, assumed to be normally distributed. The estimation method was REML and the degrees of freedom method was Kenward−Roger. Polynomial orthogonal contrasts were used to test the linear and quadratic models. Statistical significance of any main effect was declared at *p* ≤ 0.05 and tendencies were discussed at 0.05 < *p* ≤ 0.10.

## 3. Results

### 3.1. Diet Characteristics and Particle Size Distribution

The ingredient composition and chemical analysis of experimental TMRs are in Table 1. Numerically greater NDF and EE concentrations and lower ROM and starch concentrations with the replacement of human-edible feed ingredients with byproducts reflected differences in the chemical composition of human-edible feed ingredients (corn grain, barley grain and soybean meal) and byproduct ingredients (corn germ meal, corn grain screens, rice bran, barley malt sprouts and blood meal). Average dietary NDF content was on the high end of the NRC (2001) minimum recommendations of 25 to 33%, for the maintenance of suitable ruminal function.

Data on particle size distribution of forages (including corn silage and alfalfa hay) and TMRs are in Table 2. Although the proportion of particles retained on the top (19 mm) and middle (8–19 mm) sieves of the Penn State Particle Separator did not differ among treatments, the proportion of particles retained on the bottom sieve (1.18–8 mm) increased and the particles retained on the bottom pan decreased as byproduct ingredients were increasingly substituted for human-edible feed ingredients (Table 2). Consequently, pef _> 1.18_ and geometric mean particle size increased (Table 2).

### 3.2. Nutrient Intake

Average DM (22.1 kg/d) and NEL (35.2 Mcal/d) intakes did not differ among treatments (Table 3). However, EE (*p* < 0.001) and NDF (*p* < 0.001) intakes increased, whereas starch (*p* < 0.001) intake decreased linearly as byproduct ingredients replaced human-edible feed ingredients.

### 3.3. Chewing Activity

Chewing activity data are presented in Table 4. Total eating time (297 min/d, on average) and eating time per kilogram of intake (13.6 min/kg of DMI, on average) were similar among treatments (Table 4). However, ruminating time (*p* = 0.06) and total chewing time (*p* = 0.10) tended to increase linearly with increasing amounts of byproducts in the diet. Ruminating and total chewing times per kg of DM intake were not affected by dietary treatments.

### 3.4. Rumen Fermentation

Mean ruminal pH was not affected by dietary treatment (Table 5). Total rumen VFA concentration tended (*p* = 0.06) to decrease linearly with greater concentrations of byproducts in the diet. Acetate concentration increased linearly with increased substitution of human-edible feed ingredients with byproduct, whereas isobutyrate, valerate and isovalerate concentrations decreased linearly (Table 5). Propionate concentration tended (*p* = 0.05) to decrease, whereas the acetate:propionate ratio tended (*p* = 0.09) to increase linearly as byproduct ingredients were included at greater concentrations in the diet.

### 3.5. Milk Yield and Composition

Milk (43.6 kg/d, on average), protein (1.31 kg/d, on average) and lactose (2.01 kg/d, on average) yields did not differ among treatments (Table 6). Substitution of human-edible feed ingredients with byproduct linearly increased 3.5% FCM and milk fat yields (Table 6). The response of ECM (*p* = 0.05) and SCM (*p* = 0.06) tended to be linear with increasing concentrations of dietary byproduct ingredients. Milk fat concentration and fat to protein ratio increased linearly as inclusion of byproduct ingredients in the diet increased. Milk protein, lactose and MUN concentrations were similar among treatments and averaged 3.01%, 4.62% and 12.84 mg/dL (Table 6), respectively. However, NEFA, BHBA and acetone in milk increased linearly with the greater inclusion of byproducts in the diet (Table 6).

Milk fatty acid concentration data are in Table 7. Including more byproduct ingredients in the diet linearly decreased concentrations of saturated, palmitic, de novo and mixed fatty acids. However, unsaturated fatty acids, MUFA, PUFA, stearic acid, oleic acid and preformed fatty acid concentrations increased (Table 7).

### 3.6. FCE, heFCE and NFP

The feed efficiency parameters (milk yield/DMI, ECM/DMI and SCM/DMI) were not affected by dietary treatments. However, FCM/DMI tended (*p* = 0.06) to increase linearly with greater inclusion of byproducts. Human-edible feed conversion efficiency and net food production for both CP and energy linearly increased with increasing dietary byproduct concentrations (Table 8).

No effect on average back fat thickness was observed, whereas a quadratic effect on average body weight, with the highest amount for BP60, was observed.

## 4. Discussion

Sustainable and economical feeding strategies for dairy production can be compared by different methods. One of these methods is the feed conversion efficiency (kg of ECM/kg of DMI). No differences in feed conversion efficiency between the treatments were observed in this study (Table 8). However, this method accounts only for the amount fed and not what was provided. It means that it does not matter what portion of the dry matter consumed by the cow is edible for humans and what portion is inedible for humans. Another approach to compare diets from a sustainability and economic standpoint is to determine the amount of human-edible food produced (milk) per unit of human-edible feed ingredients offered [4,5,8,33,34]. Concentrates with a greater proportion of BP instead of HE feed ingredients typically have greater production efficiency for human edibles [6,9,18], in agreement with our results of greater net food production and HeFCE for both energy and protein (Table 8).

Cows fed greater amounts of BP had similar DMI and milk production as cows fed a HE diet with more cereal grains and soybean meal. Milk production is positively related to dietary starch content [35], likely due to greater provision of glucogenic precursors such as propionate [36]. However, excessive starch may compromise rumen health [37] and impair milk yield [38]. Overall, any changes in glucogenic and lipogenic precursors in the diet alters energy balance, possibly modifying milk fat content or milk yield [39]. In this study, BP-based diets had greater fat and NDF, but lower starch content, being more lipogenic. Van Knegsel et al. (2005) suggested that lipogenic nutrients could increase milk yield and milk fat percentage [39]. This agrees with findings from several studies replacing CG (corn grain), SBM (soybean meal) or both with different byproducts, such as sugar beet pulp and wheat bran [5,17], dried distiller′s grains with solubles [40] or rapeseed meal and dried distillers grains with solubles [41]. Also, the meta-analysis by Ferraretto et al. (2013) reported DMI was not altered by dietary starch content [35]. On the other hand, the highest level of dietary fat was about 6.1% in the current experiment, therefore it did not decrease DMI. According to NRC (2001), supplemental fat often decreases DMI when the total dietary fat concentration exceeds 6 to 7% of DM [29]. Due to more fat and NDF, but less starch in BP than HE ingredients, cows fed BP-based diets had greater fat and NDF intakes, but lower starch intake compared with cows fed HE.

Milk fat percentage and, consequently, 3.5% FCM and ECM increased when cows were fed BP concentrate diets in comparison with the control diet (Table 6). However, milk fat percentage, and thus fat:protein, is very low among cows in this study. This is because, in Iranian dairy farms, due to the low quality of forage and the low digestibility of NDF, a high amount of concentrate (60%), especially grains, is fed in the diet, which caused the low milk fat percentage. Previous studies in this situation, conducted at Iranian dairy farms, have also reported low milk fat percentages [10,35,42,43,44]. Byproducts differ from traditional HE concentrates in nutrient composition. Replacing HE with BP feed ingredients increased the NDF and fat content of diets and decreased the starch and ROM content (Table 1); however, EE and NDF intakes increased while starch intake reduced (Table 3). Furthermore, pef _> 1.18_ and geometric mean particle size were greater for BP-based diets (Table 2). Providing sufficient peNDF supports ruminal function as longer forage particles stimulate chewing and rumination, salivary buffer secretion and the formation of a functional ruminal digesta mat [45,46]. Maintaining sufficient dietary peNDF is important for ensuring ruminal conditions that promote efficient carbohydrate fermentation [45,46]. The effect of the ingestion of long particles on increased ruminal pH may be related to increased chewing activity and improved ruminal health and function [45]. Additionally, ruminating time tended to increase as BP replaced HE feed ingredients (Table 4), which could possibly increase the flow of saliva and rumen buffering capacity, improving rumen fermentation significantly and rumen pH numerically (Table 5). In the present study, feeding greater amounts of BP feed ingredients increased ruminal acetate concentration, which could possibly increase milk fat. Moreover, replacing starch-rich feeds with fiber-rich byproducts in dairy cow diets reduces the risk of acidosis [47]. Previously, Van Knegsel et al. (2007) reported reduced milk fat production when lipogenic ingredients were replaced with glucogenic ingredients [48]. The same study suggested that a lower supply of acetate in high dietary starch diets may contribute to lower concentrations of milk fat. In the present study, grain with BP improved milk fat content and production as well as FCE for FCM production. The improvement of milk fat was expected, as it has been reported previously [49], and increased milk fat production in the present study was supported by improved ruminal fermentation and acetate production. Indeed, acetate is one of the important precursors for de novo milk fat synthesis in the mammary gland [50,51], and improving rumen fermentation can prevent induction of milk fat depression [52].

Milk fatty acid concentration changed in this study, which is consistent with known effects of unsaturated fat supplements on de novo fatty acids synthesis in the mammary gland [53]. It is well acknowledged that feeding unsaturated oils is associated with a decrease in de novo synthesis of short chain fatty acids (SCFA) and medium-chain fatty acids (MCFA; [54,55,56]). Ney (1991) showed that the decrease in MCFA is an improvement in the profile of milk fatty acids, because MCFA form the hypercholesterolemic part of milk fat [56]. Milk fat C16:0 originates either from the diet or it is synthesized in the mammary gland [57]. The decrease in the milk fat concentration of C16:0 in cows fed the BP diets compared with the control diet indicates a decrease in the de novo synthesis of C16:0 in the mammary gland. Preformed fatty acids in milk originate from the diet or from adipose tissue mobilization [58,59] and increased concentration of these fatty acids in cows fed the BP-based diets (lower starch) compared with the control diet was likely a consequence of increased body reserve mobilization as a response to the lower energy balance, and also the higher amount of fat content in BP-based diets, which potentially consists of unsaturated fatty acids because of rice bran and corn germ meal.

## 5. Conclusions

Feeding greater amounts of byproduct-based concentrate instead of human-edible feed ingredients for high-producing dairy cows reduced human-edible inputs and increased HeFCE up to 9.22 MJ/MJ edible for energy and 4.14 kg/kg edible for protein, whereas net food production increased 15 fold for protein and 1.5 fold for energy. Furthermore, rumen fermentation was enhanced as the starch content of the diet decreased and the fat and fiber content increased, leading to a linear increase in the milk fat percentage and a yield of 3.5% FCM. Overall, feeding byproduct-based concentrate instead of human-edible feed ingredients increased net food production and improved the performance of high-producing Holstein cows. Because this was a short-term study, BW change could not be measured, and this could have changed and influenced short-term responses. Therefore, future research is warranted to evaluate these feeding strategies for longer periods.

## Figures and Tables

**Table 1:** Ingredient and chemical composition of experimental diets (DM basis) and estimated proportion of human edibles.

	Diets ^1^			Human-Edible Proportion
Item	BP26	BP60	BP95	
Ingredient composition, % of DM				
Alfalfa hay	20.00	20.00	20.00	0.0
Corn silage	20.00	20.00	20.00	0.0
Corn grain, ground	24.00	12.00	-	0.8
Barley grain, ground	3.20	1.60	-	0.8
Soybean meal, ground	12.00	6.00	-	0.8
Extruded soybean	2.00	1.00	-	0.8
Cottonseed	2.32	2.32	2.32	0.2
Corn gluten meal	2.48	2.48	2.48	0.2
Corn bran	10.80	10.80	10.80	0.2
Corn germ meal	-	6.38	12.76	0.2
Corn grain screens	-	6.00	12.00	0.2
Rice bran	-	4.00	8.00	0.2
Barley malt sprouts	-	3.50	7.00	0.2
Blood meal	-	0.80	1.60	0.0
Sodium–bicarbonate	1.00	1.00	1.00	0.0
Calcium carbonate	0.64	0.82	1.00	0.0
Dicalcium phosphate	0.48	0.24	-	0.0
Magnesium oxide	0.08	0.06	0.04	0.0
Vitamin–mineral premix ^2^	0.80	0.80	0.80	0.0
Salt	0.20	0.20	0.20	0.0
Human-edible proportion, % of DM	36.08	23.58	11.07	-
Chemical composition				
DM, % of as-fed	53.2	53.7	53.9	-
CP	16.8	16.9	16.8	-
NDF	32.1	35.3	38.2	-
Forage NDF	20.06	20.06	20.06	-
Starch	26.1	22.1	18.0	-
Ether extract	3.4	4.7	6.1	-
ROM ^3^	12.9	12.0	11.6	
NE_L_, ^4^ Mcal/kg of DM	1.62	1.61	1.62	-

^1^ Experimental diets differed by concentrate part: BP26 = concentrate containing 26% byproducts; BP60 = concentrate containing 60% byproducts; BP95 = concentrate containing 95% byproducts. ^2^ Vitamin–mineral premix contained (DM basis) 130,000 IU/kg of vitamin A; 360,000 IU/kg of vitamin D3; 12,000 IU/kg of vitamin E; 10 g/kg of Mn; 16 g/kg of Zn; 4 g/kg of Cu; 0.15 g/kg of I; 0.12 g/kg of Co; 0.8 g/kg of Fe; and 0.08 mg/kg of Se. ^3^ Residual organic matter (ROM) was calculated as %OM-%CP-%NDF-%Starch-%fat according to NASEM (2021) [20] ^4^ based on tabular values (Cornell Net Carbohydrate and Protein System; [21]).

**Table 2:** Physical characteristics of forages and diets ^1^.

	Forage		Diets ^3^		
Item ^2^	Corn Silage	Alfalfa Hay	BP26	BP60	BP95
% DM retained on sieves					
19 mm	24.6	4.0	6.2	6.4	6.3
8 mm	55.7	32.6	22.8	22.6	22.7
1.18 mm	18.9	42.4	38.6	42.4	45.3
Pan	0.85	20.9	32.4	28.6	25.7
pef _> 8_	0.80	0.37	0.29	0.29	0.29
pef _> 1.18_	0.99	0.79	0.69	0.71	0.74
Xgm, ^4^ mm	11.92	4.35	3.42	3.56	3.69
SDgm, ^5^ mm	2.07	2.84	3.13	3.05	2.97

^1^ Particle length variables were measured using the Penn State Particle Separator (The Pennsylvania State University, University Park; [25]). ^2^ pef _> 8_ and pef _> 1.18_ = physical effectiveness factor determined as the proportion of particles retained on 2 sieves [26] and 3 sieves [25], respectively. ^3^ Experimental diets differed by concentrate part: BP26 = concentrate containing 26% byproducts; BP60 = concentrate containing 60% byproducts; BP95 = concentrate containing 95% byproducts. ^4^ Geometric mean particle size, calculated according to the method of the American Society of Agricultural Engineers ([27]; method S424.1). ^5^ Geometric standard deviation of particle size, calculated according to the method of the American Society of Agricultural Engineers ([27]; method S424.1).

**Table 3:** Effect of replacing human-edible feed ingredients with byproducts on nutrient intake.

	Diets ^1^			SEM	*p* Value	
Item	BP26	BP60	BP95		Linear	Quadratic
DM, kg/d	21.84	22.22	22.17	0.61	0.37	0.49
Starch, kg/d	5.71	4.95	3.98	0.14	<0.01	0.23
EE, kg/d	0.74	1.04	1.35	0.03	<0.01	0.84
NDF, kg/d	7.01	7.84	8.47	0.21	<0.01	0.33
NE_L_, Mcal/d	35.38	36.00	35.91	0.10	0.37	0.50

^1^ Experimental diets differed by concentrate part: BP26 = concentrate containing 26% byproducts; BP60 = concentrate containing 60% byproducts; BP95 = concentrate containing 95% byproducts.

**Table 4:** Effect of replacing human-edible feed ingredients with byproducts on chewing activities.

	Diets ^1^			SEM	*p* Value	
Item	BP26	BP60	BP95		Linear	Quadratic
Eating time						
min/d	295	294	301	11	0.59	0.69
min/kg of DMI	13.70	13.37	13.72	0.73	0.98	0.48
Ruminating time						
min/d	473	467	502	16	0.06	0.12
min/kg of DMI	21.90	21.24	22.85	1.12	0.21	0.89
Total chewing time						
min/d	768	761	803	21	0.10	0.17
min/kg of DMI	35.60	34.60	36.56	1.68	0.36	0.11

^1^ Experimental diets differed by concentrate part: BP26 = concentrate containing 26% byproducts; BP60 = concentrate containing 60% byproducts; BP95 = concentrate containing 95% byproducts.

**Table 5:** Effect of replacing human-edible feed ingredients with byproducts on rumen pH and fermentation.

	Diets ^1^			SEM	*p* Value	
Item	BP26	BP60	BP95		Linear	Quadratic
Rumen pH	6.01	6.15	6.21	0.14	0.14	0.73
Total VFA, mM	114	101	102	5.49	0.06	0.23
VFA proportions, %						
Acetate	65.45	66.34	67.20	1.17	<0.01	0.98
Propionate	23.26	22.19	21.92	1.01	0.05	0.48
Isobutyrate	0.436	0.432	0.354	0.03	0.01	0.18
Butyrate	8.49	8.81	8.57	0.30	0.82	0.36
Isovalerate	1.12	1.08	0.88	0.06	<0.01	0.27
Valerate	1.25	1.15	1.08	0.01	<0.01	0.84
Acetate:propionate	2.90	3.09	3.10	0.19	0.09	0.35

^1^ Experimental diets differed by concentrate part: BP26 = concentrate containing 26% byproducts; BP60 = concentrate containing 60% byproducts; BP95 = concentrate containing 95% byproducts.

**Table 6:** Effect of replacing human-edible feed ingredients with byproducts on lactation performance.

	Diets ^1^			SEM	*p* Value	
Item	BP26	BP60	BP95		Linear	Quadratic
Yield, kg/d						
Milk	43.10	43.70	43.94	1.00	0.37	0.82
3.5% FCM ^2^	39.12	40.14	41.33	1.06	0.02	0.90
ECM ^2^	39.44	40.26	41.23	0.96	0.05	0.92
SCM ^2^	35.98	36.65	37.50	0.92	0.06	0.90
Fat	1.26	1.31	1.38	0.06	<0.01	0.70
Protein	1.30	1.31	1.32	0.03	0.76	0.96
Lactose	2.00	2.02	2.02	0.05	0.68	0.83
Composition, %						
Fat	2.95	2.99	3.13	0.13	<0.01	0.23
Protein	3.04	3.00	2.99	0.04	0.19	0.55
Lactose	4.64	4.62	4.60	0.03	0.68	0.83
Fat:protein	0.97	0.99	1.04	0.04	<0.01	0.19
MUN, mg/dL	12.65	12.84	13.05	0.45	0.25	0.97
NEFA, µeq/L	341.41	404.48	481.31	20.01	<0.01	0.60
BHBA, mmol/L	0.076	0.097	0.117	0.007	<0.01	0.82
Acetone, mmol/L	0.157	0.182	0.213	0.008	<0.01	0.73

^1^ Experimental diets differed by concentrate part: BP26 = concentrate containing 26% byproducts; BP60 = concentrate containing 60% byproducts; BP95 = concentrate containing 95% byproducts. ^2^ Yield of 3.5% FCM = 0.432 × milk yield + 16.23 × fat yield, ECM = 12.82 × fat yield + 7.13 × protein yield + 0.323 × milk yield and SCM = milk yield × [(12.24 × fat% × 0.01) + (7.1 × protein% × 0.01) + (6.35 × lactose% × 0.01) − 0.0345] as according to NRC (2001) equations.

**Table 7:** Effect of replacing human-edible feed ingredients with byproducts on milk fatty acid concentration.

	Diets ^1^			SEM	*p* Value	
Fatty Acid Concentration, g/100 g	BP26	BP60	BP95		Linear	Quadratic
Saturated fatty acids	70.90	68.04	65.81	0.54	<0.01	0.39
Unsaturated fatty acids	22.82	26.09	28.61	0.46	<0.01	0.35
MUFA ^2^	19.80	22.54	24.74	0.39	<0.01	0.41
PUFA ^3^	3.31	3.89	4.19	0.13	<0.01	0.22
Palmitic acid (C16:00)	26.24	24.14	22.71	0.45	<0.01	0.33
Stearic acid (C18:00)	15.46	16.45	17.65	0.38	<0.01	0.68
Oleic acid (C18:1, cis-9)	15.97	18.13	19.99	0.32	<0.01	0.60
De novo fatty acids	32.14	30.05	27.74	0.44	<0.01	0.76
Mixed fatty acids	28.30	25.76	24.03	0.53	<0.01	0.37
Preformed fatty acids	39.57	44.19	48.22	0.67	<0.01	0.67

^1^ Experimental diets differed by concentrate part: BP26 = concentrate containing 26% byproducts; BP60 = concentrate containing 60% byproducts; BP95 = concentrate containing 95% byproducts. ^2^ Monounsaturated fatty acids. ^3^ Polyunsaturated fatty acids.

**Table 8:** Effect of replacing human-edible feed ingredients with byproducts on feed conversion efficiency, human-edible efficiency and net food production.

	Diets ^1^			SEM	*p* Value	
Item	BP26	BP60	BP95		Linear	Quadratic
Feed conversion efficiency						
Milk yield/DMI	1.99	1.98	1.99	0.07	0.97	0.62
FCM/DMI	1.79	1.80	1.86	0.03	0.06	0.35
ECM/DMI	1.81	1.81	1.86	0.03	0.15	0.34
SCM/DMI	1.65	1.64	1.69	0.03	0.21	0.37
HeFCE ^2^						
CP, kg/kg edible	1.06	1.66	4.14	0.07	<0.01	<0.01
Energy, MJ/MJ edible	2.27	3.62	9.22	0.10	<0.01	<0.01
Net food production						
CP, kg/d	0.064	0.52	1.00	0.03	<0.01	0.70
Energy, MJ/d	62.8	83.0	104.7	2.36	<0.01	0.73
Average BW, kg	672	680	675	14	0.19	0.01
Average BFT ^3^, mm	25.75	26.50	26.58	0.69	0.31	0.64

^1^ Experimental diets differed by concentrate part: BP26 = concentrate containing 26% byproducts; BP60 = concentrate containing 60% byproducts; BP95 = concentrate containing 95% byproducts. ^2^ Human-edible feed conversion efficiency (HeFCE). ^3^ Back fat thickness was measured using ultrasonographic method [30].

## Data Availability

Data are available from the corresponding author upon request.
